# Early Events in the Pathogenesis of Foot-and-Mouth Disease in Pigs; Identification of Oropharyngeal Tonsils as Sites of Primary and Sustained Viral Replication

**DOI:** 10.1371/journal.pone.0106859

**Published:** 2014-09-03

**Authors:** Carolina Stenfeldt, Juan M. Pacheco, Luis L. Rodriguez, Jonathan Arzt

**Affiliations:** 1 Plum Island Animal Disease Center, Foreign Animal Disease Research Unit, Agricultural Research Service, United States Department of Agriculture, Greenport, New York, United States of America; 2 Oak Ridge Institute for Science and Education, PIADC Research Participation Program, Oak Ridge, Tennessee, United States of America; Friedrich-Loeffler-Institut, Germany

## Abstract

A time-course study was performed to elucidate the early events of foot-and-mouth disease virus (FMDV) infection in pigs subsequent to simulated natural, intra-oropharyngeal, inoculation. The earliest detectable event was primary infection in the lingual and paraepiglottic tonsils at 6 hours post inoculation (hpi) characterized by regional localization of viral RNA, viral antigen, and infectious virus. At this time FMDV antigen was localized in cytokeratin-positive epithelial cells and CD172a-expressing leukocytes of the crypt epithelium of the paraepiglottic tonsils. *De novo* replication of FMDV was first detected in oropharyngeal swab samples at 12 hpi and viremia occurred at 18–24 hpi, approximately 24 hours prior to the appearance of vesicular lesions. From 12 through 78 hpi, microscopic detection of FMDV was consistently localized to cytokeratin-positive cells within morphologically characteristic segments of oropharyngeal tonsil crypt epithelium. During this period, leukocyte populations expressing CD172a, SLA-DQ class II and/or CD8 were found in close proximity to infected epithelial cells, but with little or no co-localization with viral proteins. Similarly, M-cells expressing cytokeratin-18 did not co-localize with FMDV proteins. Intra-epithelial micro-vesicles composed of acantholytic epithelial cells expressing large amounts of structural and non-structural FMDV proteins were present within crypts of the tonsil of the soft palate during peak clinical infection. These findings inculpate the paraepiglottic tonsils as the primary site of FMDV infection in pigs exposed via the gastrointestinal tract. Furthermore, the continuing replication of FMDV in the oropharyngeal tonsils during viremia and peak clinical infection with no concurrent amplification of virus occurring in the lower respiratory tract indicates that these sites are the major source of shedding of FMDV from pigs.

## Introduction

Foot-and-mouth disease (FMD) is a highly contagious infection of cloven-hoofed animals, with a renowned ability of rapid transmission amongst susceptible hosts. The causative agent, FMD virus (FMDV), is a non-enveloped positive sense RNA virus belonging to the Aphthovirus genus of the Picornavirus family [1]. Outbreaks of FMD within developed countries that are normally kept free of the disease lead to immediate and severe impact upon agricultural production, with prolonged restrictions on export of animal products. Furthermore, in the large regions of the world in which FMD is endemic, the disease poses a constant threat to the health and welfare of livestock, thereby compromising the livelihood of farmers and causing instability of food supplies [2].

The characteristic clinical manifestations of FMD which include blanching and vesiculation of cornified epithelium within the oral cavity and in areas of non-haired skin, can be seen across a wide range of susceptible host-species, including domestic and wild ruminants and suids [3]–[5]. Despite several common features of the clinical infection, there are certain elements of FMDV pathogenesis that are specific for different host-species. Pigs are often recognized as being more severely affected by the clinical phase of FMD when compared to cattle and sheep [6]. However, in contrast to ruminants, pigs have proven to be more efficient in clearing the infection as there is no convincing evidence of a persistent, sub-clinical “carrier state” of FMDV in suids [5]–[7]. It has also been widely accepted that, whilst pigs are capable of generating large amounts of aerosolized virus, they are less susceptible to aerogenous infection than ruminants [8], [9]. Further evidence of critical host-specific differences in the molecular pathways of FMDV infection has been provided through evidence of a restricted host-range of FMDV strains that carry specific deletions within the 3A-coding region of the genome [10]–[12]. These viruses have proven to be significantly attenuated in cattle, whilst retaining pathogenicity in pigs [10], [12], [13].

In contrast to recently gained knowledge characterizing acute FMDV-infection in cattle [14], [15], detailed temporo-anatomical mapping of virus distribution during early phases of infection has not been fully achieved in pigs. Several experimental studies have aimed at investigating the early events of FMDV pathogenesis in pigs [16]–[20]. However, the conclusions of the published works are somewhat variable. Earlier work suggested a high susceptibility of pigs to infection through aerosol exposure in contrast to oral inoculation [18], [21], with initial virus replication found within the upper respiratory tract, before disseminating to also involve the lungs. This was later contradicted [8], [9], with more recent investigations suggesting that following initial virus entry through lymphoid tissues of the pharyngeal region, the most substantial amplification of virus takes place in the skin at secondary lesion sites [16], [17]. The variable conclusions of these previous studies can partially be explained by variations in study design with use of different inoculation routes. Furthermore, a substantial part of investigations have been based on analysis of tissues obtained from pigs that were already viremic, which precludes conclusions regarding the initial events of infection.

The aim of the current study was to elucidate the early events of FMDV pathogenesis in pigs by use of a standardized, simulated natural experimental system that has been demonstrated to efficiently and consistently infect pigs with FMDV [20]. This approach enabled localization of the primary site of FMDV replication to morphologically distinct segments of reticular epithelium within the paraepiglottic tonsils. Furthermore, it is concluded that the tonsil of the soft palate supports continued viral replication through the peak of clinical infection, thus contributing the major source of viral shedding into the environment.

## Materials and Methods

### 2.1 Virus strains

The virus used for this study was a cattle-derived FMDV A24 Cruzeiro that had been passed once in pigs. Details regarding the generation of the virus stock, including determination of 50% pig-heel infectious doses per ml (PHID_50_) have been published previously [22]. In the current study, an inoculation dose of 100 PHID_50_/pig was used based on previous experiments confirming that this dose generates a highly synchronous infection following intra-oropharyngeal (IOP) inoculation of pigs [20].

### 2.2 Animal experiments

All animal studies were performed within the BSL-3 containment facility at the Plum Island Animal Disease Center. Experimental protocols were subjected to prior approval by the facility's Institutional Animal Care and Use Committee (protocol number 231-12-R), which functions to ensure ethical and humane treatment of experimental animals. All experimental animals (castrated male Yorkshire pigs, weighing approximately 30 kg upon delivery) were obtained from the same vendor (Animal Biotech Industries Inc., Danboro, PA). Following an initial clinical examination, pigs were allowed an acclimation period of approximately 1–2 weeks prior to initiation of experiments.

Nineteen pigs were used for the studies described herein (Tables 1–2). In order to facilitate intensive monitoring of antemortem infection dynamics, as well as harvest of tissue samples at desired time points, separate animal experiments were staggered, with 2–6 pigs in separate groups subjected to the exact same experimental protocol. Four pigs were exclusively used for monitoring of antemortem infection dynamics, and were thus not included in post-mortem collection of tissue samples.

**Table 1:** Tissue distribution of FMDV during the pre-viremic phase of infection following IOP inoculation.

	6 HPI		12 HPI			% Positive		Mean GCN
*Animal ID*	**37400**	**37401**	**37896**	**37897**	**37403**	**qRT-PCR**	**VI**	
**Oral cavity/oropharynx**								
Tongue- anterior	-	-	3.04	-	-	20	0	**3.04**
Lingual tonsil	**3.48**	**3.08**	-	-	**4.96**	60	60	**3.84**
Paraepiglottic tonsil	**3.00**	**4.61**	**3.37**	**+**	**4.93**	80	100	**3.98**
Tonsil of the soft palate	-	-	**4.00**	-	-	20	20	**4.00**
**Nasal cavity/Nasopharynx**								
Dorsal soft palate -Rostral	2.56	**+**	-	-	**3.08**	40	40	**2.82**
Dorsal soft palate -Caudal	-	**+**	-	-	**4.01**	20	40	**4.01**
Nasal turbinates	-	-	-	-	-	0	0	-
Nasopharyngeal tonsil	-	-	-			0	20	-
Dorsal nasopharynx	-	-	-	**+**	**3.42**	20	40	**3.42**
**Lungs/Trachea**								
Trachea	-	-	-	-	-	0	0	-
Cranial lung	-	-	-	-	-	0	0	-
Mid lung	-	-	-	-	2.87	20	0	2.87
Caudal lung	-	-	-	-	-	0	0	-
**Additional tissues**								
Visceral organs*	-	-	-	-	-	0	0	-
Medial Retropharyngeal LN	-	-	-	-	-	0	0	-
Submandibular LN	-	-	-	-	-	0	0	-
Hilar LN	-	-	-	-	-	0	0	-
Popliteal LN	-	-	-	-	-	0	0	-
Adrenal gland	-	-	-	-	-	0	0	-
Bone marrow	-	-	-	-	-	0	0	-
Myocardium	-	-	-	-	-	0	0	-
Psoas Muscle	-	-	-	-	-	0	0	-
Semimembraneous muscle	-	-	-	-	-	0	0	-
Shoulder Muscle	-	-	-	-	-	0	0	-
Neck skin	-	-	-	-	-	0	0	-
Snout skin	-	-	-	-	-	0	0	-
Coronary band	-	-	-	-	-	0	0	-

Numbers represent Log_10_ genome copy numbers (GCN)/mg of tissue. Bold numbers indicate that samples were positive for both FMDV RNA (qRT-PCR) and virus isolation, (+) indicates that virus isolation was positive but FMDV RNA content was below the limit of detection, (-) indicates double negative samples. Limit of detection: 2.52 Log_10_ GCN/mg of tissue.

* Visceral organs included liver, spleen, kidney, pancreas, ileum and Peyer's patches. Other tissues analyzed with consistently negative results were epiglottis, larynx, pharyngeal-diverticulum, salivary glands, adrenal glands, bone marrow.

**Table 2:** Tissue distribution of FMDV during viremic phase of infection following IOP inoculation.

	24 HPI		48 HPI		78 HPI		Mean GCN
*Animal ID*	*34257*	*34256*	*34258*	*34255*	*37404*	*37405*	
*FMDV RNA copies/*µ*l in serum (log_10_)*	1.02	2.69	6.53	7.17	6.14	5.83	
**Oral cavity/oropharynx**							
Tongue	2.81	**4.63**	**5.71**	**6.46**	**6.85**	**7.08**	**5.59**
Lingual tonsil	NA	NA	NA	NA	**5.78**	**5.45**	**5.61**
Paraepiglottic tonsil	**5.71**	**6.29**	**5.59**	**5.30**	**5.91**	**5.03**	**5.64**
Tonsil of the soft palate	**+**	**+**	**4.78**	**4.22**	**6.05**	**5.95**	**5.25**
**Nasal cavity/Nasopharynx**							
Dorsal soft palate -Rostral	**3.46**	**4.87**	**5.70**	**6.09**	**5.44**	**5.03**	**5.10**
Dorsal soft palate -Caudal	**5.10**	**4.02**	**6.32**	**6.13**	**5.53**	**4.72**	**5.30**
Nasal turbinates	-	**2.80**	**4.15**	**6.28**	**5.63**	**5.09**	**4.79**
Nasopharyngeal tonsil	**2.71**	**+**	**6.21**	**6.08**	**3.89**	**4.94**	**4.77**
Dorsal nasopharynx -Caudal	**3.96**	**4.98**	**5.62**	**5.78**	**5.58**	**5.28**	**5.20**
Dorsal nasopharynx -Rostral	**4.41**	**3.35**	**6.35**	**5.67**	**6.02**	**4.92**	**5.12**
**Lungs/Trachea**							
Trachea	**+**	-	**5.69**	**6.98**	**4.66**	**5.71**	**5.76**
Proximal cranial lobe	-	**3.22**	**5.49**	**6.33**	**4.41**	**4.83**	**4.86**
Mid cranial lobe	-	**3.04**	**2.61**	**3.98**	**5.68**	**4.37**	**3.94**
Distal cranial lobe	-	**2.94**	**3.30**	**4.42**	**4.38**	**4.13**	**3.83**
Proximal mid lobe	-	**2.85**	**4.67**	**5.78**	**4.85**	**4.01**	**4.43**
Mid mid lobe	-	**3.33**	**3.74**	**5.34**	**4.80**	**4.37**	**4.32**
Distal mid lobe	-	**3.35**	**3.92**	**4.96**	**5.19**	**4.42**	**4.37**
Proximal caudal lobe	-	**2.67**	**3.87**	**6.96**	**3.83**	**4.72**	**4.41**
Mid caudal lobe	-	**3.02**	**3.87**	**5.81**	**4.22**	**4.50**	**4.28**
Distal caudal lobe	-	**3.34**	**3.93**	**5.33**	**4.81**	**4.80**	**4.44**
**Visceral organs**							
Liver	-	**+**	**+**	**4.10**	**3.45**	**4.66**	**4.07**
Spleen	-	**+**	**2.99**	**4.79**	**5.93**	**4.97**	**4.67**
Kidney	-	**+**	**3.55**	**4.06**	*NA*	*NA*	**3.81**
Pancreas	-	**+**	**3.81**	**4.52**	*-*		**4.16**
Peyer's Patches	**+**	**+**	**4.28**	**5.21**	**4.27**	**3.39**	**4.29**
**Additional tissues**							
Medial Retropharyngeal LN	**+**	**+**	**3.28**	**4.49**	**5.61**	**3.40**	**4.19**
Submandibular LN	**+**	-	**3.87**	**4.19**	**5.03**	**2.81**	**3.98**
Hilar LN	-	**+**	**3.58**	**4.84**	**5.59**	**4.71**	**4.68**
Renal LN	-	-	**2.91**	**6.36**	**6.08**	**3.67**	**4.76**
Popliteal LN	**+**	**3.81**	**5.69**	**7.78**	**6.20**	**5.18**	**5.73**
Heart -right ventricle	-	-	**5.10**	**5.99**	**4.68**	**4.23**	**5.00**
Heart- left ventricle	-	-	**4.85**	**5.46**	**6.01**	**4.16**	**5.12**
Psoas Muscle	-	-	**4.44**	**5.79**	**3.70**	**6.04**	**4.99**
Semimembraneous muscle	**+**	-	**6.04**	**6.92**	**6.87**	**5.51**	**6.33**
Shoulder Muscle	-	**+**	**5.75**	**6.32**	**5.48**	**5.23**	**5.70**
Neck skin	-	-	**4.99**	**5.61**	**6.21**	**5.38**	**5.55**
Snout skin	-	-	**5.94**	**7.00**	**8.40**	**8.07**	**7.35**
Coronary band	-	-	**9.66**	**9.91**	**7.41**	**8.46**	**8.86**

Numbers represent Log_10_ genome copy numbers (GCN)/mg of tissue. FMDV RNA content in serum is expressed as Log_10_ FMDV RNA copies/µl to facilitate comparison with viral content in tissues. Bold numbers indicate that samples were positive for both FMDV RNA (qRT-PCR) and virus isolation, (+) indicates that virus isolation was positive but FMDV RNA content was below the limit of detection, (-) indicates double negative samples. Limits of detection: tissue samples  = 2.52 Log_10_ GCN/mg, serum≤0.1 Log_10_ GCN/µl (corresponding to an assay detection limit of 2.68 Log_10_ GCN/ml of serum).

#### 2.2.1 Inoculation

Pigs were inoculated using a novel, simulated natural system of direct intra-oropharyngeal (IOP) inoculation [20]. In brief; pigs were deeply sedated using a mixture of Telazole, Ketamine and Xylazine, (3, 8 and 4 mg/kg respectively) and placed in dorsal recumbency. The inoculum consisting of 100 PHID_50_ of virus diluted to a total volume of 2 ml in minimal essential media containing 25 mM HEPES, was deposited directly onto the tonsil of the soft palate by use of a 4” stainless steel cannula. Pigs were kept with their heads in a stable position for 1 minute to ensure adequate contact between inoculum and the tonsil. As a smaller scale “proof-of-concept” study, a separate group of pigs were exposed to virus through direct contact with IOP-inoculated animals. This cohort of four pigs were housed together with two IOP-inoculated donor pigs through 24 hours. The contact exposure took place from 36 to 60 hours post inoculation (hpi) of the donors, which corresponded to the transition from pre-clinical to clinical phase of infection. The animals had free access to water during the contact period, but feed was withheld to stimulate interaction between donors and recipients. The two donor pigs were removed from the pen at the end of the 24 hour exposure.

#### 2.2.2 Clinical evaluation and antemortem sampling

A standardized protocol for sampling and clinical evaluation was followed throughout the experiments. Blood samples were collected from the jugular vein, nasal swabs were obtained using small cotton swabs, and oropharyngeal (OP) swabs were obtained through direct targeting of the tonsil of the soft palate using a larger cotton swab. Swabs were immersed in 2 ml minimal essential media containing 25mM HEPES directly upon collection. Blood samples were separated through centrifugation, and OP swabs were centrifuged in order to extract the fluid absorbed by the larger cotton swab. All samples were immediately frozen at −70°C until further processing. The progression of the clinical infection (lesion distribution) was quantitated using a previously described scoring system [20], [23]. In brief, each of 16 digits showing a characteristic FMDV lesion contributed one point towards a cumulative score, with four additional single points added for lesions within the oral cavity, on the snout, on the lower lip, and on carpal/tarsal skin, thus allowing a maximum score of 20.

In order to monitor acute phase infection dynamics and development of clinical disease, IOP-inoculated pigs were intensively sampled and clinically evaluated through the first 96 hpi. From these animals, blood samples were collected at 6 hour intervals from 0 to 24 hpi, and at 12 hour intervals subsequently. Clinical evaluations and collection of nasal and OP swabs were performed directly before and after inoculation and subsequently at 2 hour intervals from 4 to 12 hpi, at 3 hour intervals from 12 to 24 hpi, and at 12 hour intervals thereafter. Additionally, clinical examinations, including rectal temperature measurements, were performed at 4 hour intervals from 36 to 54 hpi in two IOP-inoculated animals in order to more precisely determine the time point at which vesicular lesions could first be observed. The 4 contact-exposed pigs were monitored through 48 hours post contact (hpc). Blood samples were collected at 12, 24 and 48 hpc, with clinical examinations and collection of swab samples performed at 4 hour intervals from 0 to 12 hpc, and at 24 and 48 hpc subsequently.

#### 2.2.3 Post mortem sample collection

Pigs were euthanized at 6, 12, 24, 48 and 78 hpi (see tables 1–2) by exsanguination under deep anesthesia (following intramuscular injection of Telazole, Ketamine and Xylazine at 4.5, 12 and 6 mg/kg respectively). All pigs were subjected to a standardized necropsy protocol immediately following euthanasia, with collection of up to 47 distinct tissue samples (Tables 1–3). Tissue collection was focused upon distinct and clearly defined regions of mucosal associated lymphoid tissues (MALT) from Waldeyer's ring, spanning the oro-and nasopharynx [24]. Oropharyngeal MALT tissues included the tonsil of the soft palate which comprises the majority of the ventral surface of the soft palate and thereby provides the dorsal boundary of the oropharynx [25]. The lingual tonsils are cryptless lymphoid aggregates located directly below the surface epithelium at the root of the tongue [25].The paraepiglottic tonsils consist of small paired tonsils located within a deep epithelial fold at the base of the epiglottis. Nasopharyngeal MALT samples consisted of the nasopharyngeal tonsil which is located at the rostro-dorsal margin of the nasopharynx (directly at the junction with nasal cavity) and thereby directly exposed to inhaled air. Additional MALT-rich nasopharyngeal mucosal surfaces were harvested and defined as dorsal nasopharynx (nasopharyngeal ceiling spanning from the nasopharyngeal tonsil to the opening of the esophagus), and the dorsal soft palate (nasopharyngeal floor spanning from the caudal aspect of the hard palate to the caudal aspect of the soft palate).

**Table 3:** Tissue distribution of FMDV during viremic phase of infection following contact exposure.

	24 HPI		48 HPI		Mean GCN
*Animal ID*	*37800*	*37801*	*37802*	*37803*	
*FMDV RNA copies/µl in serum (log_10_)*	**4.02**	**3.79**	**6.27**	**6.30**	
**Oral cavity/oropharynx**					
Tongue	**3.00**	**3.37**	**5.95**	**5.20**	**4.38**
Lingual tonsil	**3.07**	**4.39**	**5.33**	**5.10**	**4.47**
Paraepiglottic tonsil	**+**	**4.95**	**4.72**	**6.11**	**5.26**
Tonsil of the soft palate	-	**+**	**4.36**	**5.17**	**4.77**
**Nasal cavity/Nasopharynx**					
Dorsal soft palate -Rostral	**3.28**	**3.27**	**5.41**	**5.50**	**4.36**
Dorsal soft palate -Caudal	**3.13**	**3.47**	**5.09**	**6.05**	**4.44**
Nasal turbinates	**2.87**	3.08	**5.59**	**5.12**	**4.17**
Nasopharyngeal tonsil	**+**	**+**	**5.33**	**6.14**	**5.74**
Dorsal nasopharynx -Caudal	**3.65**	**3.52**	**5.28**	**5.79**	**4.56**
Dorsal nasopharynx -Rostral	**3.38**	**2.83**	**5.81**	**5.72**	**4.44**
**Lungs/Trachea**					
Trachea	**+**	2.51	**5.68**	**4.84**	**4.34**
Proximal cranial lobe	**+**	**3.31**	**5.66**	**4.82**	**4.60**
Mid cranial lobe	**+**	**3.35**	**5.46**	**4.20**	**4.34**
Distal cranial lobe	**+**	3.44	**5.43**	**4.67**	**4.51**
Proximal mid lobe	**+**	**3.05**	**5.40**	**5.42**	**4.62**
Mid mid lobe	**+**	3.99	**5.57**	**4.82**	**4.79**
Distal mid lobe	**+**	**3.54**	**5.38**	**4.53**	**4.48**
Proximal caudal lobe	-	**3.55**	**5.97**	**5.05**	**4.86**
Mid caudal lobe	**+**	**3.40**	**5.76**	**5.28**	**4.81**
Distal caudal lobe	**+**	**3.58**	**5.51**	**5.21**	**4.77**
**Visceral organs**					
Liver	**+**	-	**4.84**	**3.85**	**4.34**
Spleen	**+**	-	**5.24**	**4.16**	**4.70**
Pancreas	**+**	-	**2.60**	**2.57**	**2.58**
Peyers Patches	**+**	-	**4.32**	**+**	**4.32**
**Additional tissues**					
Medial Retropharyngeal LN	**+**	**3.77**	**6.66**	**3.48**	**4.64**
Submandibular LN	**+**	**+**	**3.28**	**4.25**	**3.77**
Hilar LN	**+**	**2.96**	**5.59**	**4.22**	**4.26**
Renal LN	**2.81**	**+**	**6.62**	**4.19**	**4.54**
Popliteal LN	**+**	**2.72**	**7.30**	**5.30**	**5.11**
Heart -right ventricle	-	-	**4.30**	**3.78**	**4.04**
Heart- left ventricle	-	-	**5.09**	**3.64**	**4.37**
Psoas Muscle	**2.85**	3.01	**5.37**	**5.24**	**4.12**
Semimembraneous muscle	**3.05**	**3.61**	**5.61**	**4.02**	**4.07**
Shoulder Muscle	-	**3.62**	**5.35**	**4.45**	**4.47**
Neck skin	**3.13**	**4.49**	**4.85**	**4.15**	**4.16**
Snout skin	**2.87**	**2.81**	**9.27**	**5.03**	**5.00**
Coronary band	**4.56**	**4.46**	**8.97**	**9.30**	**6.82**

Numbers represent Log_10_ genome copy numbers (GCN)/mg of tissue. FMDV RNA content in serum is expressed as Log_10_ FMDV RNA copies/µl to facilitate comparison with viral content in tissues. Bold numbers indicate that samples were positive for both FMDV RNA (qRT-PCR) and virus isolation, (+) indicates that virus isolation was positive but FMDV RNA content was below the limit of detection, (-) indicates double negative samples. Limits of detection: tissue samples  = 2.52 Log_10_ GCN/mg, serum≤0.1 Log_10_ GCN/µl (corresponding to an assay detection limit of 2.68 Log_10_ GCN/ml of serum).

Each tissue sample was divided into three 30 mg aliquots which were placed in individual tubes before being frozen on liquid nitrogen. An adjacent specimen was collected from each tissue sample, that was embedded in optimal cutting temperature media (Sakura Finetek, Torrance, CA) and frozen above a bath of liquid nitrogen. Frozen tissue samples were transferred to the lab within two hours after collection, and were stored at −70°C until further processing.

### 2.3 FMDV RNA detection

Two aliquots of each tissue sample collected at necropsy were individually thawed and macerated using a TissueLyser bead beater (Qiagen, Valencia, CA). 50 µl of tissue macerate was transferred to 96-well plates, and samples were subjected to qRT-PCR analysis following the protocol described below.

**Figure 1:**
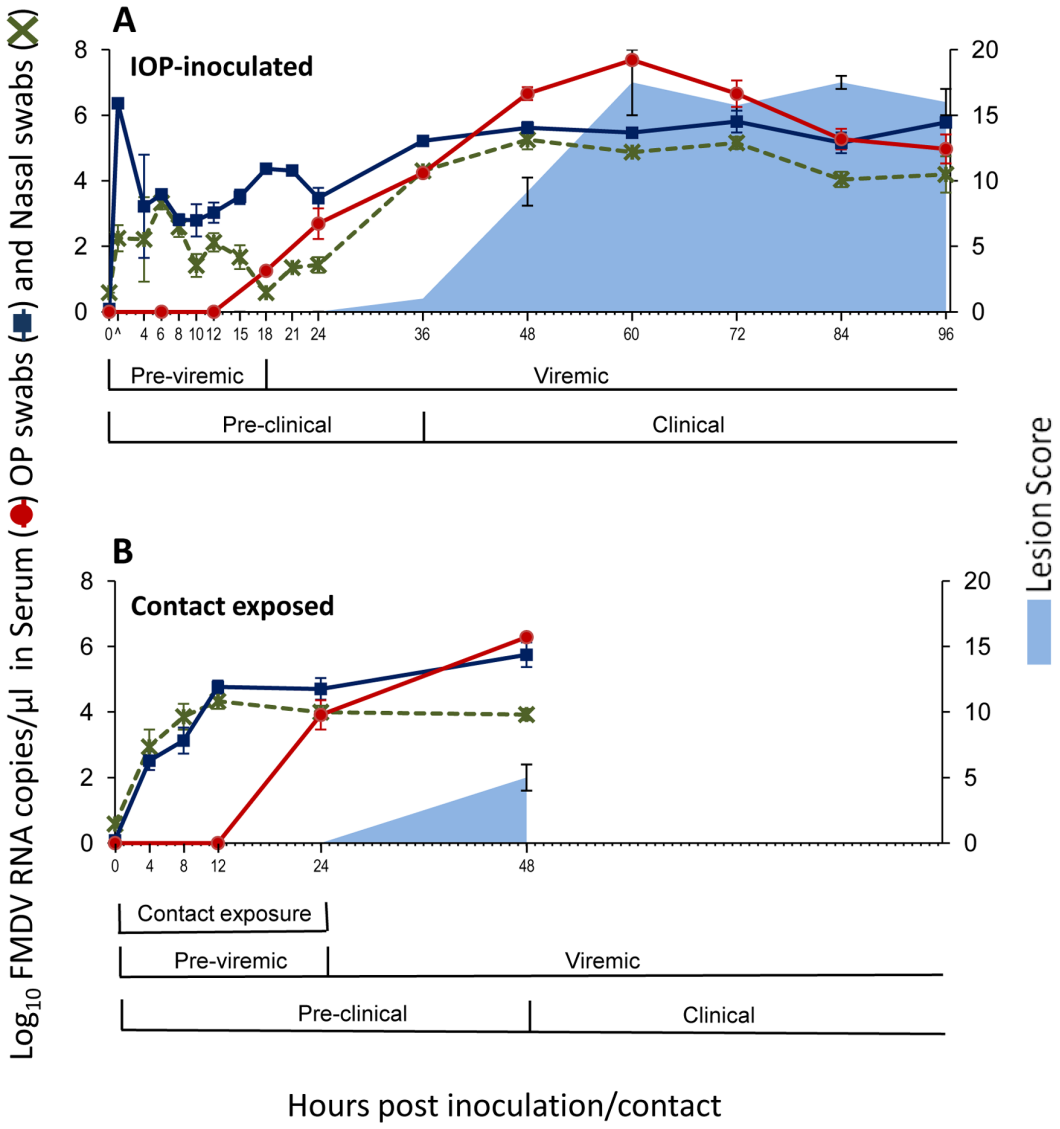
Antemortem infection dynamics in pigs infected with FMDV A24 Cruzeiro through intra-oropharyngeal inoculation (A; n = 15) or contact exposure (B; n = 4). The four contact exposed pigs were housed together with two IOP-inoculated donor pigs from 0 to 24 hpc, corresponding to 36 to 60 hpi for the donors. FMDV RNA detection in serum, nasal and tonsil swabs was performed through qRT-PCR, and is presented as Log_10_ genome copy numbers (GCN)/µl. Data presented are average values (mean +/−SEM) based on samples collected from all pigs included at each time point. “∧” on time scale in figure 1A indicates swab samples harvested directly following inoculation.

Tissue macerates, serum and swabs were analyzed using qRT-PCR, targeting the 3D region of the FMDV genome [26] as previously described [14], [15]. Cycle threshold values were converted into FMDV genome copy numbers (GCN) per milligram or microliter, by use of standard curves based on analysis of 10-fold dilutions of in-vitro synthesized FMDV RNA. The equations of the curve of RNA copy numbers versus Ct values were further adjusted for average mass of tissue samples and specific dilutions used during processing of samples. The qRT-PCR results reported in Tables 1–2 are the higher GCN/mg value of the 2 samples processed per tissue per animal. Results reported in Figure 1 represent the mean (+/−SEM) log_10_ GCN/ml for all animals sampled at each time point.

### 2.4 Virus isolation

Aliquots of macerated tissue samples and fluid extracted from OP swabs (see descriptions above) were cleared from debris and potential bacterial contamination by centrifugation through Spin-X filter columns (Costar Cat.no 8163). Tissue macerates, tonsil swabs and sera were subsequently analyzed for infectious FMDV through virus isolation (VI) on LFBK αvβ6 cells [27], [28], following a protocol previously described [15]. Presence of FMDV was further confirmed by qRT-PCR analysis of VI cell culture supernatants.

### 2.5 Immunomicroscopy

Following screening for contents of FMDV RNA using qRT-PCR, subsequent detection of antigen in cryosections was performed by immunohistochemistry (IHC) and multichannel immunofluorescence (MIF) as previously described [14], [29]. Slides were examined with a wide-field, epifluorescent microscope, and images were captured with a cooled, monochromatic digital camera. Images of individual detection channels were adjusted for contrast and brightness and merged in commercially available software (Adobe Photoshop CS6). Additional negative control tissue sections were prepared from corresponding tissues derived from a non-infected pig. Non-structural FMDV (3D) protein was detected using a mouse monoclonal antibody F19-6 [30] and FMDV structural protein (VP1) was detected using mouse monoclonal antibody 6HC4 [31]. MIF experiments included labeling of phenotypic cell markers using the following antibodies: rabbit anti-cytokeratin (Life technologies 180059), mouse anti-porcine SLA class II DQ (AbD Serotec MCA 1335), mouse anti-porcine CD8 (AbD Serotec MCA 1223), mouse anti-cytokeratin peptide 18 (Sigma Aldrich C1399) and mouse anti- CD172a (Washington State University, DH59B).

### 2.6 Statistical analysis

Differences in positive FMDV detection between distinct tissue samples obtained from pre-viremic pigs were compared using a 2-tailed Z-test for proportions [32].

## Results

### 3.1 Clinical progression following IOP inoculation and contact exposure

The clinical progression of FMD was highly synchronous across IOP-inoculated and contact exposed pigs that were kept alive long enough to develop clinical disease. In both IOP inoculated and contact exposed pigs, there was a consistent reduction in the activity level of the pigs observed at12 hours post exposure (hpe) when the animals became less responsive to external stimuli and more reluctant to rise. This change in clinical condition progressed through to 24 hpe, when pigs were moderately obtunded and would not rise unless strongly encouraged. Vesicular lesions were first detected in the coronary bands of the hind feet, and were observed as early as 36 hpe in one animal. At 48 hpe, all animals had coronary band vesicles on one or more feet, and rectal temperatures above 40°C. At this time point, the majority of animals were severely obtunded, with markedly reduced responses to external stimuli. Despite only moderate vesiculation at the coronary bands, several pigs were unable or unwilling to rise and/or to support their body weight while standing. The most severely affected individuals were euthanized due to animal welfare concerns. Lesion scores progressed substantially from 48 to 96 hpe (Figure 1A), with additional vesicles developing on accessory digits, in the oral mucosa, on the snout and on the skin covering the tarsal and carpal joints. Coronary band vesicles spread to include the solar epithelium of the heel bulbs which was sloughed and eventually replaced with thick, disorganized scar tissue in the animals that survived the acute phase of infection.

### 3.2 Antemortem infection dynamics

#### 3.2.1. Infection dynamics following IOP inoculation

High levels of FMDV RNA, interpreted as residual inoculum, were consistently detected in OP swabs collected immediately following IOP inoculation (Figure 1A). Detection in OP swabs decreased during the initial 8 hpi, after which tonsil shedding gradually increased from 12 hpi through the onset of clinical infection (at 36 to 48 hpi) to subsequently maintain stable levels through the clinical phase of infection (Figure 1A). Although FMDV RNA was also detected in nasal swabs directly following inoculation and through the pre-viremic phase of infection, the magnitude of detection was lower than in OP swabs, and levels did not rise considerably until after the onset of viremia. This difference in viral RNA levels between OP and nasal swabs decreased at the onset of clinical disease, with only marginally lower RNA quantities detected in nasal swabs through the clinical phase of infection (Figure 1A). FMDV RNA and infectious virus could be detected in serum from 18 hpi. There was an increase in viral RNA levels in serum until peak viremia was reached at 60 hpi, after which levels gradually declined (Figure 1A). Isolation of infectious FMDV from antemortem samples correlated almost perfectly with results from qRT-PCR detection. Of all samples examined, only two specimens were negative in virus isolation but positive for RNA detection through qRT-PCR.

#### 3.2.2 Infection dynamics following contact exposure

Infection dynamics in the 4 contact-exposed pigs were monitored following a less intensive sampling approach. These animals cohabitated with 2 IOP-inoculated donors for a period of 24 hours (0–24 hpc in Figure 1B; 36 to 60 hpi of donors), representing the transition from pre-clinical to clinical infection in the donors. There was a gradual increase in the levels of FMDV RNA detected in tonsil and nasal swabs in the contact exposed pigs during the first 12 hpc, after which tonsil detection continued to increase whilst nasal shedding decreased (Figure 1B). These pigs were viremic (both FMDV RNA and virus isolation positive) at 24 hpc, with a continued increase of viral RNA levels in serum in the 2 pigs that were kept alive until 48 hpc (Figure 1B). Contact-exposed pigs were euthanized at 24/48hpc since the purpose of this experiment was to compare the infection dynamics and tissue distribution of virus between IOP and contact-exposed pigs during the early phase of infection.

### 3. 3 Tissue distribution of FMDV

#### 3.3.1 FMDV tissue distribution following IOP inoculation

Pigs were euthanized at pre-determined time points regardless of the progression of the clinical infection. Post mortem examinations included collection of up to 47 distinct tissue samples, with minor variations in the tissue list depending on the expected phase of disease at the time of euthanasia (Tables 1–3). In the two pigs that were euthanized at 6 hpi, only the lingual- and paraepiglottic tonsils had uniformly consistent detection of both FMDV RNA and infectious virus (Table 1). The dorsal soft palate contained low amounts of FMDV RNA in one of the two pigs, and infectious virus (without concurrent RNA detection) was detected at this site in the other pig. It is noteworthy that no virus or viral RNA was detected in the tonsil of the soft palate at this time point, despite this tonsil constituting the anatomical site most directly targeted during inoculation. At 12 hpi, FMDV was detected by virus isolation in all 3 pigs' paraepiglottic tonsils. This tissue was also positive for viral RNA in two out of three pigs. Additionally, FMDV RNA was detected in the caudo-dorsal nasopharynx and proximal mid lung from one of the three pigs, whilst FMDV infectivity was restricted to the pharyngeal region, with detection in lingual, paraepiglottic and nasal tonsils, the dorsal soft palate and dorsal nasopharynx. At this time point, FMDV RNA and infectivity could be detected in the tonsil of the soft palate in one out of three pigs (Table 1).

In order to identify the site(s) of primary viral replication, the prevalence of virus detection, for both qRT-PCR and virus isolation, was determined across all pre-viremic pigs (5 pigs euthanized at 6 and 12 hpi). The highest detection prevalence was found in the paraepiglottic tonsil, with FMDV RNA detected in 4 out of 5 pigs (80%), and infectious virus in all 5 pigs investigated (100%). The detection prevalence for both viral RNA and infectious virus was 60% for the lingual tonsil, whilst the corresponding prevalence for the tonsil of the soft palate was 20%. The differences in proportions of positive detection were statistically significant between the paraepiglottic tonsil and the tonsil of the soft palate (p = 0.037 for RNA-detection; p = 0.0047 for virus isolation).

There was marked dissemination of virus distribution coincident with detection of viremia and appearance of vesicular lesions (Table 2). The two pigs that were euthanized at 24 hpi had comparatively low levels of FMDV RNA in serum (1.02 log_10_ RNA copies/µl and 2.69 log_10_ RNA copies/µl, for pigs 257 and 256, respectively; see table 1). Neither of these pigs had clinical FMD lesions. There was a substantial difference in the tissue distribution of virus in these two pigs, with viral detection largely restricted to pharyngeal tissues in one of the animals (pig 257), whilst FMDV RNA and infectivity was also detected in all pulmonary sites in the second 24 hpi pig (pig 256; Table 2). For both of the IOP-inoculated pigs euthanized at 24 hpi, the highest measured FMDV RNA quantities were detected in the paraepiglottic tonsils.

Animals euthanized at 48 and 78 hpi were highly viremic (Figure 1A; Table 2). In these pigs, infectious virus and viral RNA were detected in essentially all tissues analyzed, including pharyngeal, pulmonary and visceral sites (Table 2). Due to the high levels of virus present in serum during peak viremia, it is difficult to discern if detection of virus in distinct anatomic sites is indicative of local viral replication or merely a result of detection of FMDV within the intravascular compartment. FMDV RNA quantities exceeding the levels measured in serum during advanced stages of viremia were only detected at lesion predilection sites including coronary bands, tongue and snout skin. The highest virus contents were recovered from coronary band lesions (up to 9.91 log_10_ GCN/mg).

#### 3.3.2 FMDV tissue distribution following contact exposure

Two contact-exposed pigs were euthanized at 24 and 48 hpc respectively. As with IOP-inoculated pigs, all 4 pigs euthanized at these timepoints were viremic. Also similar to IOP inoculated pigs, the animals that were euthanized at 24 hpc had no clinical FMD lesions. However, serum concentration of virus in pigs euthanized at this time point was 1 to 3 log_10_ RNA copies/µl higher in the contact exposed pigs than in pigs subjected to IOP inoculation. In contact exposed pigs euthanized at 24 hpc, infectious FMDV was also isolated from a broader distribution of tissues compared to the corresponding time following IOP inoculation (Table 2). These two contact-exposed pigs had substantial variation in distribution of FMDV RNA detection, similar to that observed between the two IOP-inoculated pigs euthanized at 24 hpi. Viral RNA detection was largely restricted to pharyngeal sites in one of the pigs (pig 800), whilst FMDV RNA was found in both pharyngeal and pulmonary tissues of the second pig (pig 801). Similar to IOP-inoculated pigs, FMDV RNA and infectivity was detected in almost all tissue samples collected from the two contact exposed pigs that were euthanized at 48 hpc. In general, FMDV RNA detection followed similar distribution patterns following the two exposure routes, with detection being largely restricted to pharyngeal and pulmonary tissues at 24 hpc, to then disseminate to also include visceral organs during peak viremia and clinical infection.

### 3.4 Microscopic localization of antigen

Throughout pre-viremic and viremic phases of infection, microscopic localization of FMDV antigen was restricted to morphologically characteristic segments of oropharyngeal tonsillar crypt epithelium. These regions of reticular-type epithelium [33] consisted of rarefied layers of epithelial cells interspersed by large numbers of mixed mononuclear leukocytes and lacked clearly defined basement membranes.

**Figure 2:**
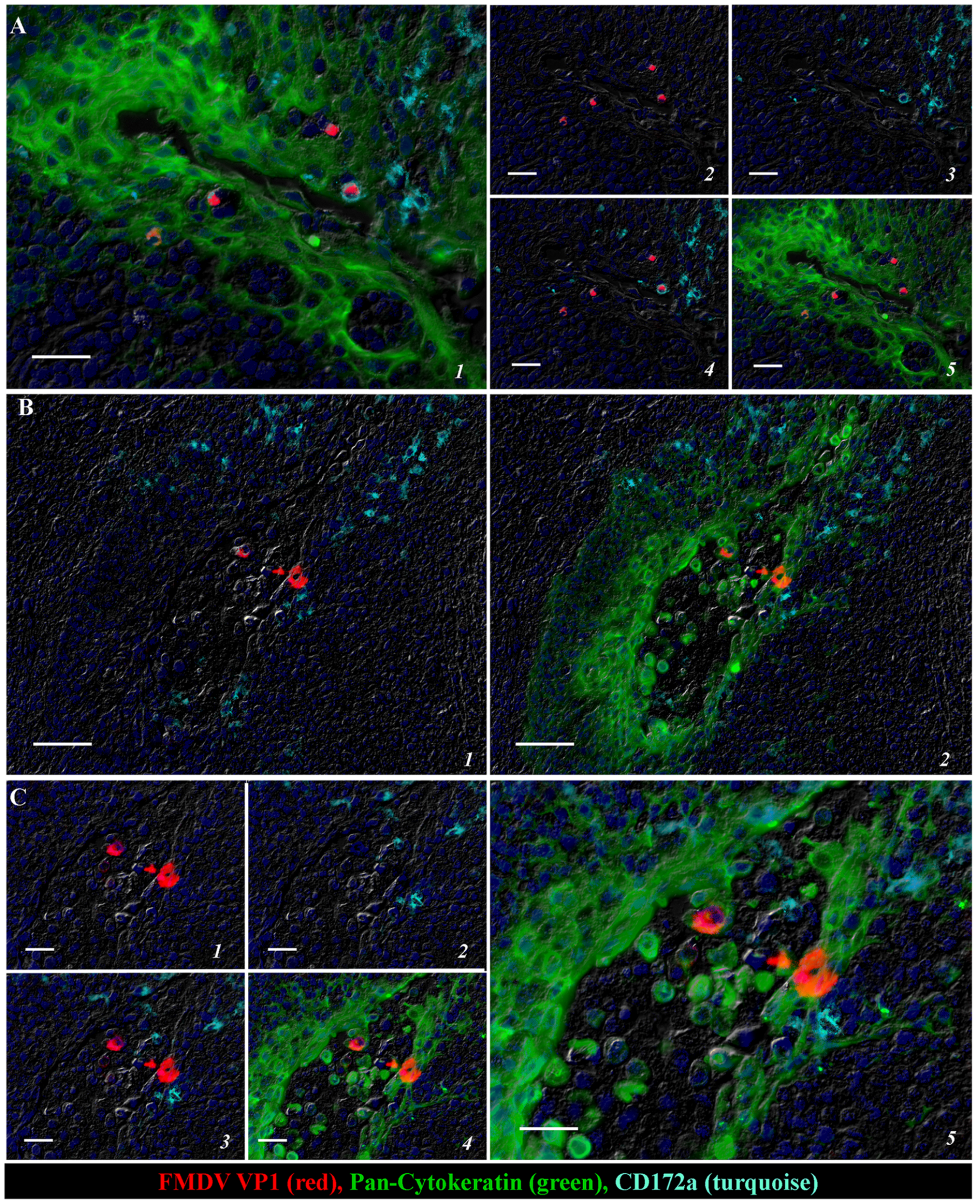
Multichannel immunofluorescent detection of FMDV capsid protein (VP1) in porcine paraepiglottic tonsils at 6 hpi (A) and 12 hpi (B–C). **A)** At 6 hpi FMDV VP1 is localized to individual cells within reticular crypt epithelium of the paraepiglottic tonsil. Virus antigen (red) is detected within few cytokeratin-positive epithelial cells (green) and CD172a-expressing non-lymphoid leukocytes (turquoise). 40× magnification, scale-bar 25 µm. **B and C)** At 12 hpi, foci of multiple FMDV VP1-positive cells are detected within similar regions of reticular crypt epithelium of the paraepiglottic tonsil. Detection of virus antigen (red) is restricted to cytokeratin-positive epithelial cells (green) in close proximity of CD172a-expressing leukocytes (turquoise). B: 20× magnification, scale bar 50 µm. C: 40× magnification, scale bar 25 µm.

At 6 hpi, FMDV structural protein VP1 (Figure 2A) and non-structural protein 3D (not shown) were localized to few individual cells scattered within reticular crypt epithelium of the paraepiglottic tonsils. At this early time point, in contrast to later time points investigated, FMDV proteins were detected within a small subset of CD172a-expressing leukocytes in addition to cytokeratin-positive epithelial cells (Figure 2A). At 12 hpi, small aggregates of FMDV-positive cells were found within similar anatomic regions (Figure 2B–C), but solely within cytokeratin-positive cells. CD172a-expressing cells were found in close proximity to infected epithelial cells, but without colocalization.

**Figure 3:**
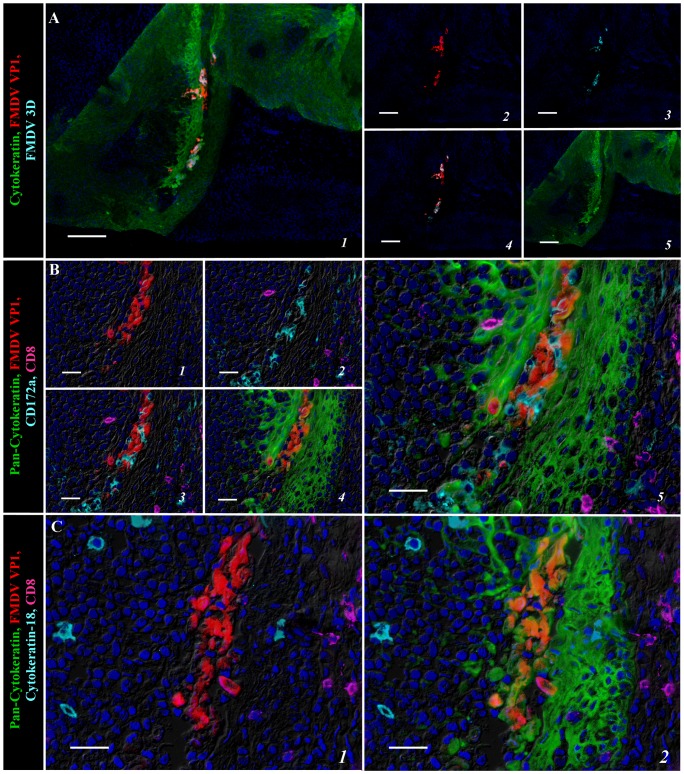
Multichannel immunofluorescent detection of FMDV structural (VP1) and non-structural (3D) protein in porcine paraepiglottic tonsils at 24 hpi. A) FMDV VP1 (red) and 3D (turquoise) proteins co-localize with cytokeratin (green) in regionally expanding foci of primary FMDV infection within reticular crypt epithelium of the paraepiglottic tonsil. 10× magnification, scale bar 100 µm. B) Serial section of region identified in (A). Localization of FMDV VP1(red) is restricted to cytokeratin-positive epithelial cells (green); leukocytes expressing CD172a (turquoise) and CD8 (purple; presumptive NK cells) are interspersed amongst virus-infected cells. 40× magnification, scale bar 50 µm. C) Serial section of region identified in (A–B). Cytokeratin-18 expressing M-cells (turquoise) localized within segments of epithelium containing FMDV VP1-positive cells (red), but without co-localization. 40× magnification, scale bar 50 µm.

At 24 hpi, corresponding to a phase of early viremia but prior to appearance of vesicular lesions, larger clusters of epithelial cells expressing both structural (VP1) and non-structural (3D) FMDV proteins were detected within crypts of the paraepiglottic tonsil (Figure 3A). Detection of viral proteins was restricted to cytokeratin-positive cells, whilst leukocyte populations expressing CD172a and CD8 were found interspersed amongst virus-infected cells, but without co-localization (Figure 3B). Cytokeratin-18 positive cells (M-cells) were also localized within epithelium segments containing FMDV-positive cells, but viral proteins were not detected within these cells (Figure 3C).

**Figure 4:**
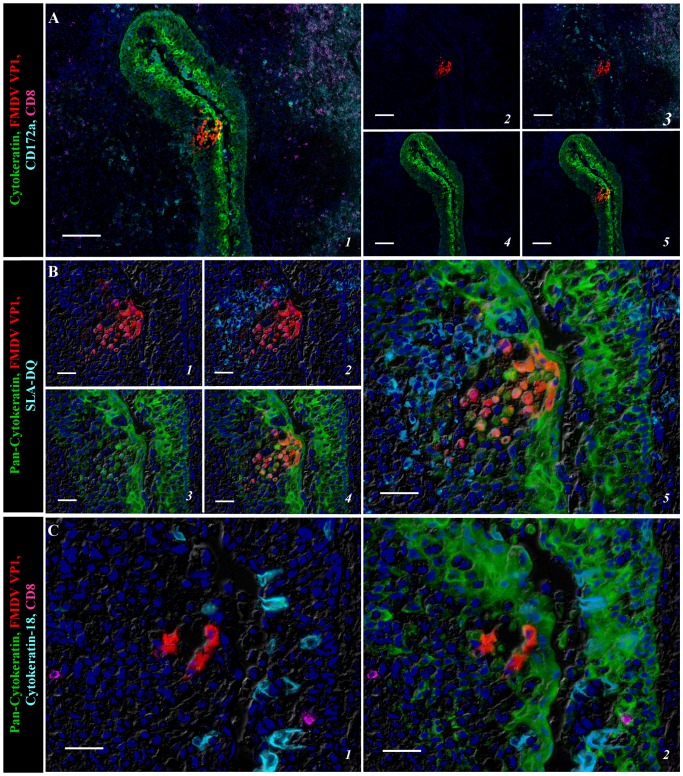
Multichannel immunofluorescent detection of FMDV structural (VP1) protein in the tonsil of the soft palate at 48 hpi. **A)** Cluster of FMDV VP1(red) positive epithelial cells (green) in a developing microvesicle within reticular crypt epithelium of the tonsil of the soft palate. Leukocytes expressing CD172a (turquoise) and CD8 (purple; presumptive NK cells) are interspersed within crypt epithelium and are present in larger numbers in adjacent (sub-epithelial) tissue. 10× magnification, scale bar 100 µm. **B)** Serial section of region identified in (A). Intraepithelial microvesicle spanning basal and spinous layers of crypt epithelium. FMDV VP1(red)/cytokeratin (green) double-positive cells are present within the vesicle and surrounding epithelium with MHC II(turquoise)-expressing cells in close proximity. 40× magnification, scale bar 25 µm. **C)** Serial section of region identified in (A–B). Cytokeratin-18 (turquoise) expressing M-cells are localized within crypt epithelium in close proximity of FMDV VP1(red) positive epithelial cells (green), but without co-localization. 40× magnification, scale bar 25 µm.

**Figure 5:**
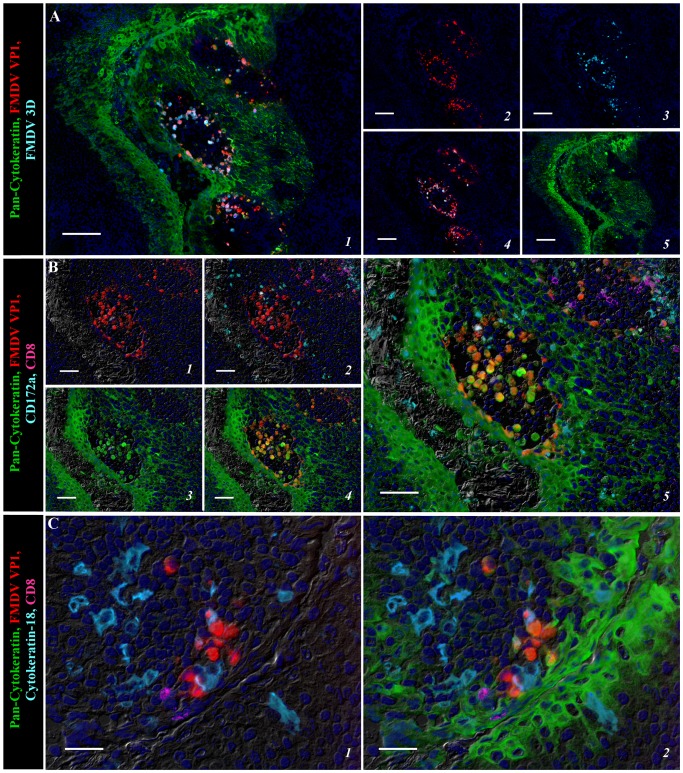
Regionally extensive micro-vesiculation within epithelial crypts on the tonsil of the soft palate at 78 hpi detected by multichannel immunofluorescence. **A)** Intra-epithelial microvesicles containing large quantities of FMDV structural (VP1; red) and non-structural (3D; turquoise) protein co-localized with cytokeratin (green) positive epithelial cells. 10× magnification, scale bar 100 µm. **B)** Serial section of region identified in A. FMDV VP1 protein (red) detected within cytokeratin (green) positive epithelial cells encircling an intra-epithelial microvesicle with acantholytic FMDV VP1/cytokeratin double positive cells detected within the vesicle lumina. CD172a (turquoise) and CD8 (purple; presumptive NK cells) leukocytes detected within and around the epithelial lesion, but without co-localization. 20× magnification, scale bar 50 µm. **C)** Cytokeratin-18 (turquoise) expressing M-cells in close proximity of FMDV VP1 (red) infected cytokeratin (green) positive epithelial cells within the tonsil of the soft palate. 40× magnification, scale bar 100 µm.

At 48 and 78 hpi, during high titer viremia and peak clinical infection, structural and non-structural FMDV proteins were detected in segments of reticular crypt epithelium in the tonsil of the soft palate, that were morphologically similar to the regions in which virus was detected in paraepiglottic tonsils at earlier timepoints. At these times, FMDV antigens were no longer detectable in paraepiglottic tonsils. Through these later time points, there was a progressive development of intra-epithelial microvesicles expanding through the basal layers of crypt epithelium. Individual microvesicles at 48 hpi (Figures 4A–B) coalesced to form regionally extensive clusters at 78 hpi (Figures 5A–B). Similar to 24 hpi, detection of viral protein was still restricted to cytokeratin-positive epithelial cells, with CD172a, CD8 and/or MHC II positive cells detected in close proximity of virus-infected cells (Figures 4–5). Acantholytic cells that were double-positive for viral proteins and cytokeratin were found within vesicle lumina (Figures 5B). Similar to earlier time points, cytokeratin-18 positive cells were found close to virus positive cells, but without co-localization (Figures 4C, 5C).

Despite repeated IHC-screening of large quantities of tissue sections derived from other organs including lungs, skin, lymph nodes and visceral organs intracellular FMDV protein was not detected in any tissues other than the tonsil of the soft palate and peripheral vesicular lesions during peak clinical infection.

## Discussion

The specific events defining the early pathogenesis of FMD in pigs have remained elusive despite several investigations targeting the subject. Previous works have provided valuable insight into the infection dynamics of FMDV in pigs [16], [17]. However, detailed and comprehensive investigations characterizing the initial stages of infection in this species have been lacking. The study described herein combines time-intensive monitoring of infection dynamics in live animals, with detailed temporo-anatomic mapping of virus distribution in tissues through the early phases of infection. A recently optimized system of intra-oropharyngeal inoculation [20] was compared to virus challenge via contact exposure, with the finding that both systems led to highly comparable antemortem infection dynamics and tissue distribution of virus.

In pre-viremic pigs, detection of FMDV by qRT-PCR and virus isolation was restricted to the pharynx. It is noteworthy that, despite inoculum being deposited directly onto the tonsil of the soft palate, the detection prevalence of virus in pre-viremic pigs was significantly higher in the paraepiglottic tonsils when compared to the tonsil of the soft palate (p-values 0.037 and 0.0047 for RNA-detection and virus isolation respectively). The paraepiglottic tonsils are small aggregates of tonsillar follicles located bilaterally within a mucosal fold at the base of the epiglottis [34]. Similar to the tonsil of the soft palate and the lingual tonsils, the paraepiglottic tonsils are exposed to material and pathogens entering through the oral route. The very early (6 hpi) detection of FMDV at this site indicates a critical role for the paraepiglottic tonsil in the early pathogenesis of FMD in pigs.

Microscopically, the earliest detectable event in FMD pathogenesis in pigs was immunolocalization of FMDV antigen to the crypt epithelium of the paraepiglottic tonsils at 6 hpi. Individual cells containing FMDV structural and non-structural proteins were found within distinct regions of reticular epithelium within tonsillar crypts. At this early stage of infection, in contrast to later time points, FMDV antigens were also identified in a small subset of cells expressing CD172a, which is a surface marker of non-lymphoid leukocytes including both neutrophils and several classes of antigen-presenting cells of monocyte lineage [35]. Detection of FMDV antigen within these cells may indicate phagocytosis of virions leading to purely degradational pathways. FMDV is generally believed to replicate only in epithelial cells; however, some works have demonstrated interactions between FMDV and dendritic cells including microscopic co-localization *in situ*, as well as evidence of these cells supporting low levels of FMDV replication *in vitro* [14], [36], [37]. Thus, it is possible that the transient intra-epithelial co-localization of FMDV within CD172a-expressing cell populations may represent uptake of FMDV by antigen presenting cells as a function of the early host response to infection. Alternatively, it is also possible that early infection of these cells is an important step in the propagation or dissemination of primary infection. Overall, the limited scope of FMDV-CD172a co-localization detected in the current investigation precludes definitive determination of the significance of this finding to the pathogenesis of FMDV. At 12 hpi, and all subsequent times, CD172a positive cells were detected in close proximity of virus infected cells, but without co-localization with virus.

Pigs euthanized at 24 hpi were in the early phase of viremia, but had not yet developed vesicular lesions. Viral distribution determined by qRT-PCR and virus isolation showed a general pattern of viral detection in pharyngeal tissues (oropharynx and nasopharynx) with additional detection in the lungs in a subset of animals. It is noteworthy that in the two IOP-inoculated pigs euthanized during this early stage of viremia, the FMDV RNA contents of the majority of pharyngeal tissues sampled, including the paraepiglottic tonsils, exceeded viral RNA levels in serum. The consistently high quantities of FMDV detected at 24 hpi in IOP-inoculated pigs further support the role of the paraepiglottic tonsil in the early pathogenesis of FMD in pigs.

Immuno-microscopy of tissues harvested at 24 hpi confirmed presence of intracellular FMDV in paraepiglottic tonsils by detection of both structural (VP1) and non-structural (3D) FMDV protein in crypt epithelium. At this stage of infection, larger foci of FMDV-positive cells had spread laterally and superficially within the crypt epithelium, but without causing consistent structural disruption of the tissue. Cytokeratin-18 positive epithelial cells were consistently detected within the tonsillar crypt epithelium, but without co-localization with viral antigen. Cytokeratin-18 is a phenotypic marker of M-cells; a sub-population of specialized epithelial cells that are involved in active uptake of luminal antigen for subsequent presentation by the host immune system [38]. Apart from an active role in antigen uptake and presentation, these cells have also been described as preferred ports of entry utilized by a large variety of pathogens [39]. However, despite the seemingly favorable anatomic location and physiological capability of these of cells, there was no evidence of active involvement of M-cells in the early events of FMDV infection in pigs.

Pigs euthanized at 48 and 78 hpi were highly viremic and were at the peak of shedding and progression of the clinical FMD infection. At these time points serum FMDV RNA contents were similar to or higher than levels measured in the majority of sampled tissues, with exception of vesicular lesions. The highest mean quantities of FMDV RNA in tissues were found in coronary band skin, which have also previously been identified as sites supporting substantial viral amplification [16], [17]. Despite IHC-screening of extensive amounts of tissue sections from various sites including lungs, non-lesion skin, lymphoid tissues and visceral organs, intracellular FMDV proteins were during this phase of infection exclusively detected within peripheral vesicular lesions, as well as in crypt epithelium of the tonsil of the soft palate. This finding contrasts a previous publication which reported ubiquitous detection of FMDV RNA by in-situ hybridization, in non-lesion skin obtained from pigs euthanized during early and late viremia [19]. Overall, the lack of detection of additional sites of FMDV replication in any pigs examined herein suggests that viremia and release of FMDV into the environment (shedding) are maintained through replication in the oropharynx and/or lesion sites.

FMDV causes a lytic infection with substantial vesiculation of cornified epithelium at sites of secondary replication [16], [17], whilst grossly detectable vesicles are usually not found at primary infection sites. Macroscopic lesions were not observed in any pharyngeal tissues inspected at post-mortem examinations in the current study. However, histological and immuno-microscopical analysis of frozen tissue sections provided convincing evidence of the occurrence of micro-vesicles within crypt epithelium of the tonsil of the soft palate during the clinical phase of infection (Figures 4–5). FMDV structural and non-structural proteins were detected in epithelial cells encircling the vesicles, with acantholytic FMDV/cytokeratin double-positive cells present within vesicle lumina. This provides the first documentation of vesicular lesions at non-classical lesion sites in pigs. Despite the relatively high contents of FMDV RNA found in a wide range of tissues sampled during viremia, micro-vesicular lesions were only detected within the crypts of the tonsil of the soft palate.

Microscopic erosions with morphologic characteristics similar to the tonsillar micro-vesicles described in the current study have previously been detected at sites of early FMDV infection in the bovine nasopharynx [14]. These separate findings demonstrate that, despite differences in the anatomic locations of primary FMDV infection in cattle and pigs, there are striking similarities in the physiological mechanisms and host-pathogen interactions that occur at the primary replication sites. The tissues that have been identified as sites of primary FMDV infection in cattle and swine respectively, also share common micro-anatomical features, such as the characteristic segments of reticular-type epithelium [33] found in close association with mucosal associated lymphoid tissue (MALT). Additionally, the early pathogenesis in pigs included a sequence of events involving distinct regions in different tissues within the oropharynx with initial viral replication occurring in the deeper paraepiglottic tonsils followed by subsequent detection in the tonsil of the soft palate, which is more directly exposed to the oropharyngeal lumen. This is similar to cattle wherein the earliest event was infection within deep epithelial crypts of the nasopharynx followed by later detection of in superficial cryptless domes of the nasopharyngeal MALT [14].

However, there are also clear differences between the output of the current work and what is known about the early pathogenesis of FMDV in cattle. The current localization of the primary site of FMDV infection to the oropharynx of pigs is consistent with the conventional wisdom that pigs are more susceptible to FMDV infection via the oral route in contrast to cattle, which are more likely to become infected via the respiratory route. The precise mechanisms that dictate these differences are complex and, surely, multifactorial; however, there are distinct anatomical and physiological differences between the upper respiratory and gastrointestinal tracts of cattle and pigs which may be contributing factors. Paraepiglottic tonsils, demonstrated herein as the earliest site of infection in pigs, do not exist in cattle. Interestingly, sheep have paraepiglottic tonsils and recent work in our laboratory suggests that they are involved in early pathogenesis of FMD in that species (Arzt et al, unpublished). Additionally, the nasopharyngeal mucosa of pigs tends to have less MALT and more stratified squamous epithelium compared to cattle (Stenfeldt and Arzt, unpublished) which may explain the apparent decreased tropism of FMDV for this region in pigs. Lastly, it may also be relevant that rumination, with repeated regurgitation of acidic rumen contents into the bovine oral cavity could decrease the viability of pH-sensitive FMDV, thus favoring infection at nasopharyngeal sites with more stabile pH.

FMDV replication in the bovine nasopharynx (primary infection site) was found to decrease concurrent to establishment of viremia, whilst increasing viral replication was simultaneously detected within the lungs [14]. In the current study, despite screening of a large amount of pulmonary tissue samples by qRT-PCR and immuno-microscopy, there was no evidence of FMDV replication in the lungs of infected pigs. It has been demonstrated that pigs are capable of excreting large amounts of aerosolized FMDV [4], [40], and previous studies have concluded that a substantial proportion of virus excreted during advanced stages of infection is derived from the lower respiratory tract [21]. The current discovery of FMDV-positive vesicular lesions within the tonsil of the soft palate clearly demonstrates that this tissue is capable of supporting high levels of viral replication through the clinical phase of infection. The anatomic location of tonsil of the soft palate on the ventral surface of the soft palate means that it is not exposed directly to air passing through the upper respiratory tract. However, air exhaled through the mouth, as would be the case e.g. during vocalization, would pass directly over the surface of this tonsil. The finding of sustained FMDV replication within the tonsil of the soft palate, together with lack of evidence supporting concurrent FMDV replication in other regions of porcine respiratory tract (lungs, trachea, nasopharynx, larynx), strongly suggests that FMDV replication within the porcine oropharynx is the most important event in the production of infectious aerosols by pigs.

## Conclusions

The overall conclusions of the work presented herein are that after infection via the upper gastrointestinal tract (oropharynx), primary FMDV replication in pigs takes place within reticular epithelium in distinct regions of oropharyngeal tonsils, with consistent predilection for the paraepiglottic tonsil. During viremia and clinical infection, substantial viral amplification occurs at secondary lesion sites with the highest viral loads found in coronary band vesicles. However, oropharyngeal tonsillar epithelium supports continued virus replication through the clinical phase of infection without detectable virus replication in the respiratory tract. Thus, it is concluded that sustained virus replication within porcine oropharyngeal tonsils, but not the lungs, contributes a major source of contagion from FMDV-infected pigs.
